# Development of an indirect ELISA to specifically detect antibodies against African swine fever virus: bioinformatics approaches

**DOI:** 10.1186/s12985-021-01568-2

**Published:** 2021-05-05

**Authors:** Zhan Gao, Jun-Jun Shao, Guang-Lei Zhang, Su-Dan Ge, Yan-Yan Chang, Lei Xiao, Hui-Yun Chang

**Affiliations:** State Key Laboratory of Veterinary Etiological Biology, OIE/National Foot-and-Mouth Disease Reference Laboratory, Lanzhou Veterinary Research Institute, Chinese Academy of Agricultural Sciences, No. 1 Xujiaping, Yanchangbao, Chengguan District, Lanzhou, 730046 Gansu China

**Keywords:** African swine fever virus, Indirect enzyme-linked immunosorbent assay, Recombinant protein, Multi-epitope

## Abstract

**Background:**

African swine fever (ASF), characterized by acute, severe, and fast-spreading, is a highly lethal swine infectious disease caused by the African swine fever virus (ASFV), which has caused substantial economic losses to the pig industry worldwide in the past 100 years.

**Methods:**

This study started with bioinformatics methods and verified the epitope fusion protein method's reliability that does not rely on traditional epitope identification. Meanwhile, it will also express and purify the constructed genes through prokaryotic expression and establish antibody detection methods.

**Results:**

The results indicated that the protein had good reactivity and did not cross-react with other swine diseases. The receiver-operating characteristic analysis was performed to verify the determination. The area under the receiver-operating characteristic curve was 0.9991 (95% confidence interval 0.9973 to 1.001).

**Conclusions:**

It was proved that the recombinant protein is feasible as a diagnostic antigen to distinguish ASFV and provides a new idea for ASFV antibody detection.

## Introduction

ASF is an acute, highly contagious swine infection caused by ASFV, with a fatality rate of up to 100% [1]. It has also resulted in substantial economic losses in the pig industry in affected areas. The World Animal Health Organization (OIE) included ASF in the list of notifiable animal diseases. ASF was first discovered in Kenya, Africa, in 1921. Before 1957, ASF outbreaks occurred only in Africa and subsequently spread to Europe and the Americas; But, except for Sardinia, the outbreak was under control. In 2007, ASF was introduced into Georgia and then through Georgia to Armenia, Azerbaijan, Russia, and other countries [2]. Furthermore, in August 2018, it was introduced into China for the first time, where it is a significant animal disease that needs to be prevented and controlled. No commercial vaccines are currently available; Thus, it is of great significance to establish an effective detection method for the prevention and treatment of ASF.

ASFV has a regular hexagonal shape with a diameter of about 200 nm. Plus, the virus particle comprises five parts: the nucleoid, core shell, inner envelope, capsid, and external envelope, from the inside to the outside. It encodes 54 structural proteins and more than 100 non-structural proteins [3]. In the face of such a complicated virus, traditional immunological test methods are undoubtedly time-consuming and laborious. Bioinformatics is an interdisciplinary field, and computers can draw rules and insights from the stored large amounts of biological, immunological test data through simulation and analogy. In this way, when the genome or protein sequence information of a new virus is obtained, the computer can analyze the virus's characteristics and the pathogen's critical epitopes based on past "experience". It can significantly reduce blindness in the experiment process.

To study whether the bioinformatics method can be used as a biological tool worthy of our trust, we predict the epitope of ASFV and use specific tests to prove whether it meets the expected effect. As a result, this study tried to predict the P30 and P54 proteins' main epitopes directly and create artificially synthesized genes with a small molecular weight and are easy to express; we named the diagnostic antigen m35. All selected epitopes are conserved sequences in the Chinese strains 2018AnhuiXCGQ, DBLN2018, and PigHLJ2018 and the standard ASF strain Georgia 2007/1. We expressed the fusion gene in the prokaryotic expression system, analyzed the recombinant protein's immune characteristics by Western blot, and then established an indirect enzyme-linked immunosorbent assay (ELISA) method based on the protein, offering an effective way for the timely diagnosis of ASFV.

## Materials and methods

### Computer prediction of epitopes

The sequences of p30 and p54 proteins of ASFV (GenBank: MK128995.1) were obtained from NCBI (http://www.ncbi.nlm.nih.gov). B cell epitope is mainly played in viral serum testing, so four online epitope prediction tools DNAStar, ABCpred Prediction (http://crdd.osdd.net/raghava/abcpred/ABC_submission.html), Scratch (http://scratch.proteomics.ics.uci.edu/) and IEDB (http://www.iedb.org/) were applied to screen the most immunogenic B cell epitopes. Since characterization of the peptide-binding specificity of swine leukocyte antigen (SLA) class I is critical to the adaptive immune response of the porcine infectious disease, it is of great significance to screen the screening of the original epitope. Here, we use NetMHCpan BA 4.1 to predict the binding affinity of peptides in proteins.

### Construction and predicted characteristics of multi-epitope m35 protein

The epitopes are linked together by the linker "GGGGS" and added to its 3 'end the 6 × His tag, whose codons were optimized for pET-28a (+), was synthesized by Nanjing Genscript. Protein secondary structure was determined using PROTEAN (DNASTAR software) and PSIRED (http://bioinf.cs.ucl.ac.uk/psipred/) [4]. It provides information on connection analysis, folding recognition, structure modeling, function prediction, protein imbalance prediction, and query sequence domain prediction.

### Expression and purification of recombinant protein

The synthetic recombinant plasmid was transformed into E. coli BL21 (Sangon Biotech Co., Ltd Shanghai China). The transformed E. coli BL21 cells were grown by adding a small amount of Luria–Bertani (LB) in a shaker at 220 rpm and 37 °C and then inoculated into a large bottle of LB (containing 30 μg/ml kanamycin) at a dilution of 1:100. After 3 h, 1 mM isopropyl β-d-1-thiogalactopyranoside (IPTG) (Solarbio, Beijing, China) was added to induce protein synthesis for 4 h. The bacteria were collected by centrifugation. The pellet was suspended in a binding buffer (0.5 M NaCl, 50 mM Tris, and 5 mM imidazole; pH 8.0) for sonication. Centrifuge the lysate after sonication and resuspend the pellet (in the form of inclusion bodies) in a binding buffer containing 8 M urea, dissolve and centrifuge at 4 °C overnight, and purify the His-tag-containing recombinant protein by affinity chromatography using NiSepharose TM excel (GE). The purified protein was refolded in dialysate (0.25 M NaCl, 50 mM Tris, 0.5 Mm EDTA, 2 mM GSH and 0.2 mM GSSG; pH8.0) at 4 °C, and contained different concentrations of urea (6 M to 0 M in decreasing order of 1 M). Finally, the protein was dialyzed twice in PBS (pH 7.4) to obtain a fully refolded protein, and the protein concentration was measured with the BCA protein detection kit (Takara) [5].

### SDS-PAGE, western blot analysis

Separate the same amount of protein using 12% SDS-PAGE electrophoresis. After completion, the gel was stained with Coomassie Blue R250 or transfer to a 0.45 μm polyvinylidene fluoride (PVDF: Millipore, USA) membrane for Western blot analysis. The primary antibodies were mouse anti-His monoclonal antibody (mAb; Abcam, MA) and ASFV positive serum.

To put it in a nutshell, the transferred membrane was blocked for 1 h at 37 °C in PBST containing 5% skim milk, and then the membrane was incubated overnight with diluted mAb or positive serum at 4 °C. The membrane was washed 3 times with PBST and incubated with diluted goat anti-mouse and goat anti-pig conjugated horseradish peroxidase (HRP; Abcam) secondary antibodies at 37 °C for 1 h. The membrane was washed 3 times with PBST, and a signal was generated using diaminobenzidine (DAB; Solarbio, Beijing, China).

### iELISA

Coated 96-well microtiter plate with gradient concentration recombinant protein (Corning, USA) and placed at 4 degrees Celsius. Incubate overnight. After washing 3 times with PBST, blocking with 5% skimmed milk powder at 37 °C for 2 h, and then washing 3 times, incubated with the positive serum of different dilutions (100μL per well) at 37 °C for 1 h, then wash the wells 3 times, and dilute anti-swine IgG antibody to 1: 20,000, add 100 μL per well, incubate for 30 min at 37 °C. After washing 3 times with PBST, 100 μL of TMB substrate was added to each well. After reacting at 37 °C for 15 min, 100 μL per well of stop solution (2 M H_2_SO_4_) was added. All samples were simultaneously measured OD at 450 nm using an ELISA microplate reader (BioTek, USA). And set up positive and negative controls.

After establishing the optimal coating concentration and dilution concentration, we tested 56 negative serum samples to determine the Cut-off value.

$${\text{Cut-off}} = {\overline{\text{X}}} + 3{\text{SD}}$$

Dot plot and receiver-operating characteristic (ROC) were performed while using the GraphPad Prism version 7.0 for Windows.

293 serum samples from Professional laboratory in Lanzhou Veterinary Research Institute, Chinese Academy of Agricultural Sciences, Lanzhou, Gansu, China:

1. 215 serum samples of negative controls were obtained from pig farms in 2015.
2. 78serum samples of ASFV positive were obtained from the Regional Laboratory of ASF, Lanzhou Veterinary Research Institute. Positive samples include 9 ASF carrier pigs that had recovered entirely from acute infection with ASFV.

### Cross-reaction experiment of ELISA

To validate the cross-reactivity, this m35 indirect ELISA was utilized to test porcine serum positive for other swine pathogens, including pseudorabies virus (PRV), porcine reproductive and respiratory syndrome virus (PRRSV), porcine circovirus (PCV), porcine epidemic diarrhea virus (PEDV), porcine delta coronavirus (PDCoV), classical swine fever virus (CSFV), and foot-and-mouth disease virus (FMDV). The positive sera were kept in our laboratory, State Key Laboratory of Veterinary Etiological Biology, Lanzhou Veterinary Research Institute. Each sample was repeated in triplicate.

### Identification of B cell epitopes

We used the ELISA method to detect the reactivity of each epitope in the recombinant protein. The polypeptide composed of the recombinant protein and other predicted dominant epitopes was sent to GenScript for synthesis. The synthesized polypeptide was used as the coating antigen to identify the epitope by positive serum Initial identification. The steps are completed following the instructions of the Takara Peptide Coating Kit.

## Result

### Construction of multiple epitope genes

The Chinese strain 2018/AnhuiXCGQ genome's genebank is MK128995.1. The results of using ABCpred Prediction, Scratch, and IEDB are shown in Table 1. According to the number of protein sequences in different threshold stages in the method, we set the Scratch threshold to 0.78, ABCpred Prediction to 0.87, and the threshold of IEDB to 0.500. The analysis results of SLA-1 are shown in Table 2, and the selected allele type is SLA-1 * 0401, which is one of the most frequently found alleles in different kinds of pigs [6]. We choose 9mers peptides with potential binding SLA‐1*0401 [7], and all selected peptides had a NetMHCpan rank score of < 2% [8]. Remove non-conservative sequences such as 153–158 in P30 by BLAST. The epitope we finally selected was repeated more, a high score in three prediction methods, and better peptide-SLA-1 binding ability in the NetMHCpan peptide prediction algorithm, as shown in Table 3.

**Table 1:** Prediction of B cell epitope of ASFV structural protein p30 and p54

Protein	Scratch			ABCpred prediction			IEDB		
	Start position	Sequence	Score	Start position	End position	Score	Start position	End position	NO
p30	173	PLKEEE	0.92724943	155	174	0.93	15	24	1
	172	TPLKEE	0.88402353						
	153	APDFNK	0.88343295	65	80	0.90	31	35	2
	72	GYTEHQ	0.88222533						
	173	PLKEEEK	0.87789045	140	153	0.89	63	84	3
	172	TPLKEEE	0.84681755						
	115	TSSFET	0.81093245	175	194	0.88	92	117	4
	114	CTSSFE	0.80846832						
	113	ECTSSF	0.80761785	146	165	0.87	120	136	5
	73	YTEHQAQ	0.79960905						
	114	CTSSFET	0.79736268	75	92	0.87	145	162	6
	19	RSSSQV	0.78848895						
	71	QGYTEH	0.7840783	134	151	0.87	164	181	7
	73	YTEHQA	0.78333067						
P54	173	TYTHKD	0.91484526	157	174	0.93	5	24	1
	145	PAEPYT	0.83470635	48	61	0.90			
	144	HPAEPYT	0.81938788	128	147	0.88			
	173	TYTHKDL	0.81866632	5	20	0.88	54	122	2
	155	QNTASQ	0.81150261	2	13	0.87			
	174	YTHKDLE	0.80539209	76	89	0.87			
	146	AEPYTT	0.79759747	65	78	0.87	132	180	3
	172	NTYTHK	0.79226505	159	174	0.87			
	174	YTHKDL	0.78092143	140	155	0.87			

Scratch, ABCpred prediction: the higher the score, the more likely the epitope, the IEDB orders the epitope from most likely to least likely

**Table 2:** Peptide candidates for SLA‐1*0401 affinity analysis (low consensus score = good binder)

Protein	Start–end position	Sequence	NetMHCpan predicted% rank score
p30	25–33	VVFHAGSLY	0.13
	111–119	ETNECTSSF	0.2
	143–151	KTVQHIEQY	0.23
	52–60	KTLLSTVKY	0.24
	115–123	CTSSFETLF	0.29
	79–87	AQEEWNMIL	1.4
	7–15	ISMKMEVIF	1.7
p54	61–69	AIEEEDIQF	0.19
	2–10	DSEFFQPVY	0.94
	18–26	LSPVTTPSF	1.3
	73–81	YQDQQWVEV	1.3
	142–150	APAHPAEPY	1.5
	155–163	TQNTASQTM	1.5
	161–169	QTMSAIENL	1.7

**Table 3:** B cell epitope sequences selected in p30 and p54

Protein	Start–end position	Sequence
p30	15–28	FKTDLRSSSQVVFH
	24–33	QVVFHAGSLY
	73–80	GYTEHQAQ
	115–123	CTSSFETLF
	173–181	TPLKEEEKE
	182–193	VVRLMVIKLLKK
P54	17–26	CLSPVTTPSF
	145–161	HPAEPYTTVTTQNTASQ
	160–169	SQTMSAIENL
	167–181	ENLRQRNTYTHKDLE

The epitope with a good score and more conservatism was finally selected through four prediction methods

### Predicted characteristics of m35 protein

**Fig. 1:**
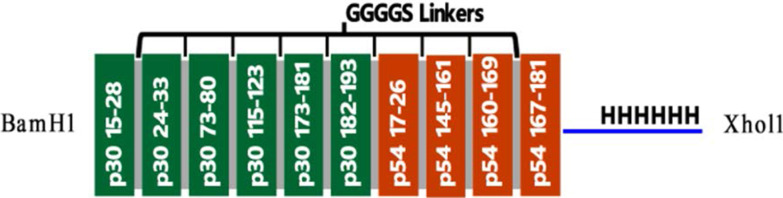
Schematic presentation of the recombinant protein. "GGGGS" is the linker. A 6x-His tag is added at the Carboxy terminus for purification and identification purposes. The enzyme dugs at both ends are BamH1 and Xhol1, respectively

**Fig. 2:**
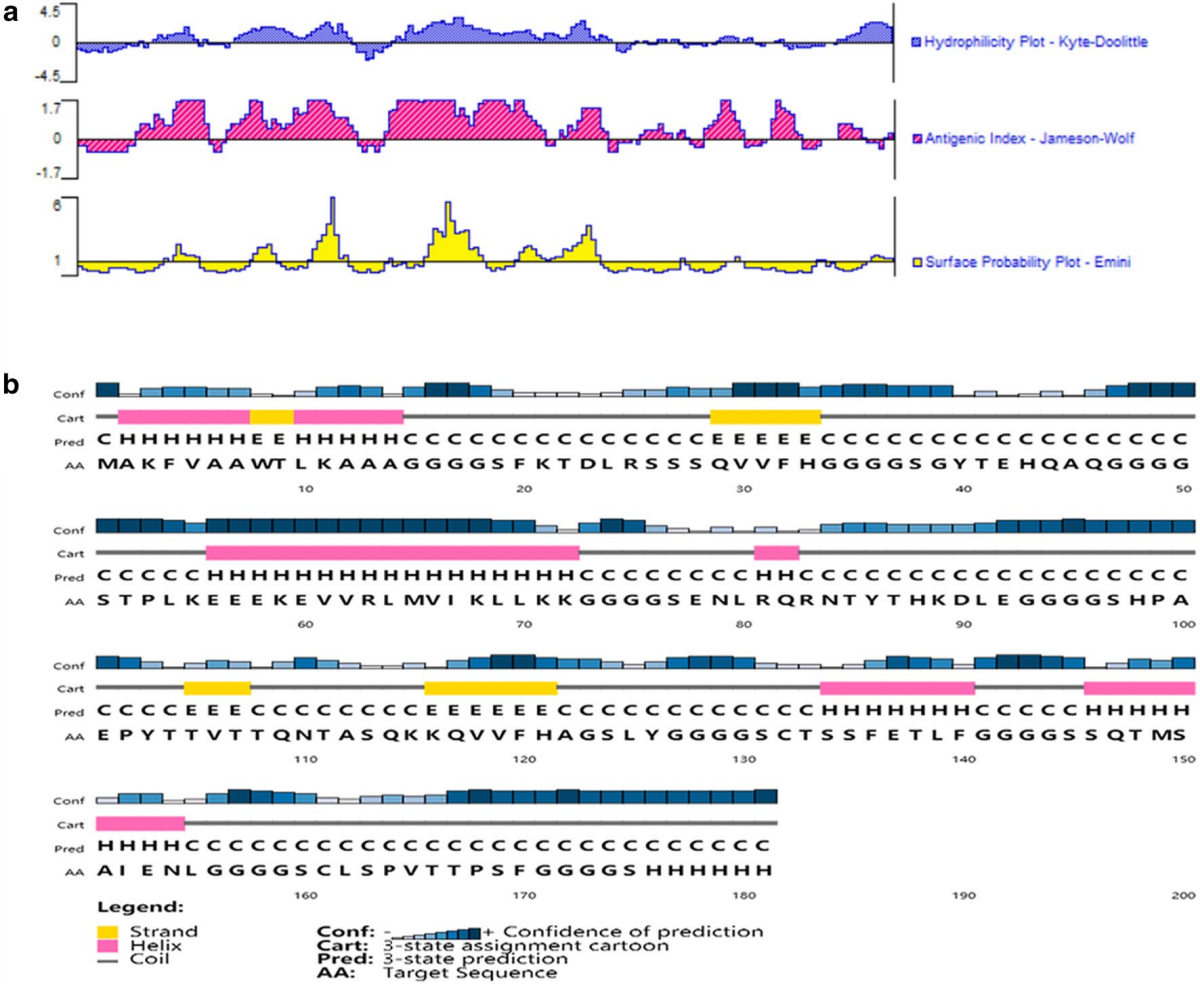
Bioinformatics prediction analysis of m35 protein. **a** Protean predicted the physical and chemical properties of proteins. **b** PSIPRED was used to predict the secondary structure of the protein

To avoid the emergence of new epitopes, the authors added the "GGGGS"linker in the middle of each epitope (Fig. 1). Through the Protean program (Fig. 2) to predict the antigenic index, hydrophobicity, and surface probability, it can be observed that the protein has a certain degree of hydrophilicity and a high antigenic index. Predictive analysis of secondary structure by PSIRED shows the presence of four alpha helices (α1, 2–14; α2, 56–72; α3, 134–140; α4, 146–154).

### Recombinant protein expression, purification and identification

**Fig. 3:**
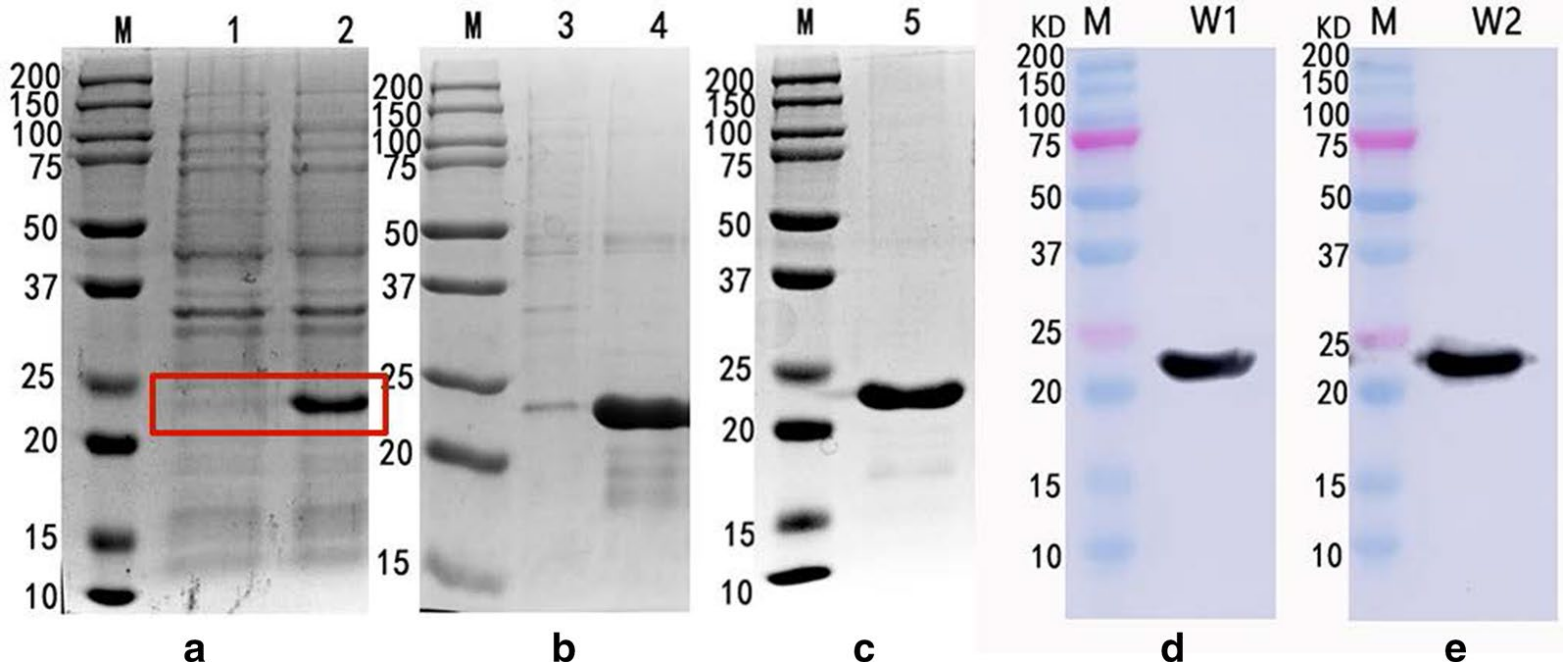
Preparation of recombinant protein. SDS-PAGE analysis of m35 protein **a** (M, marker; Lane 1, uninduced cells; Lane 2, IPTG-induced cells for 4 h) **b** (M, marker; Lane 3, the supernatant of IPTG-induced cells; Lane 4, deposition of IPTG-induced cells) **c** (M, marker; Lane 5, purified recombinant protein) Western blotting analysis of the purified recombinant protein using the mouse anti‐His monoclonal antibody (mAb) (**d**) and the ASFV-positive serum (**e**)

A synthetic 558-bp gene was synthesized by optimizing E. coli expression codons by Nanjing GenScript. Then the gene sequence was inserted into the bacterial expression vector pET-28a and transformed into E. coli BL21 (DE3) cells (Fig. 3). After induction, sonication, and analysis by SDS-PAGE, it can be seen that the recombinant protein is expressed. Select Ni–NTA affinity purification to obtain the purified product. Eventually, the authors obtained 1 ml of m35 protein solution with a concentration of 1.4 mg/ml. The immunoreactivity of m35 protein was checked by Western blot. We can see that the recombinant protein can react with HIS monoclonal antibody and ASFV positive serum, respectively.

### iELISA

The results of the checkerboard titration method are shown in Table 4.When the positive serum OD value is around 1 and the P/N value is relatively large, the conditions are optimal (shown in the red wire frame).so we choose the best coating concentration to be 0.25 μg/mL, the best serum dilution concentration is 1:100, and the determination results of 56 negative sera are shown in Table 5, calculated by the formula cut-off value is 0.3380.

**Fig. 4:**
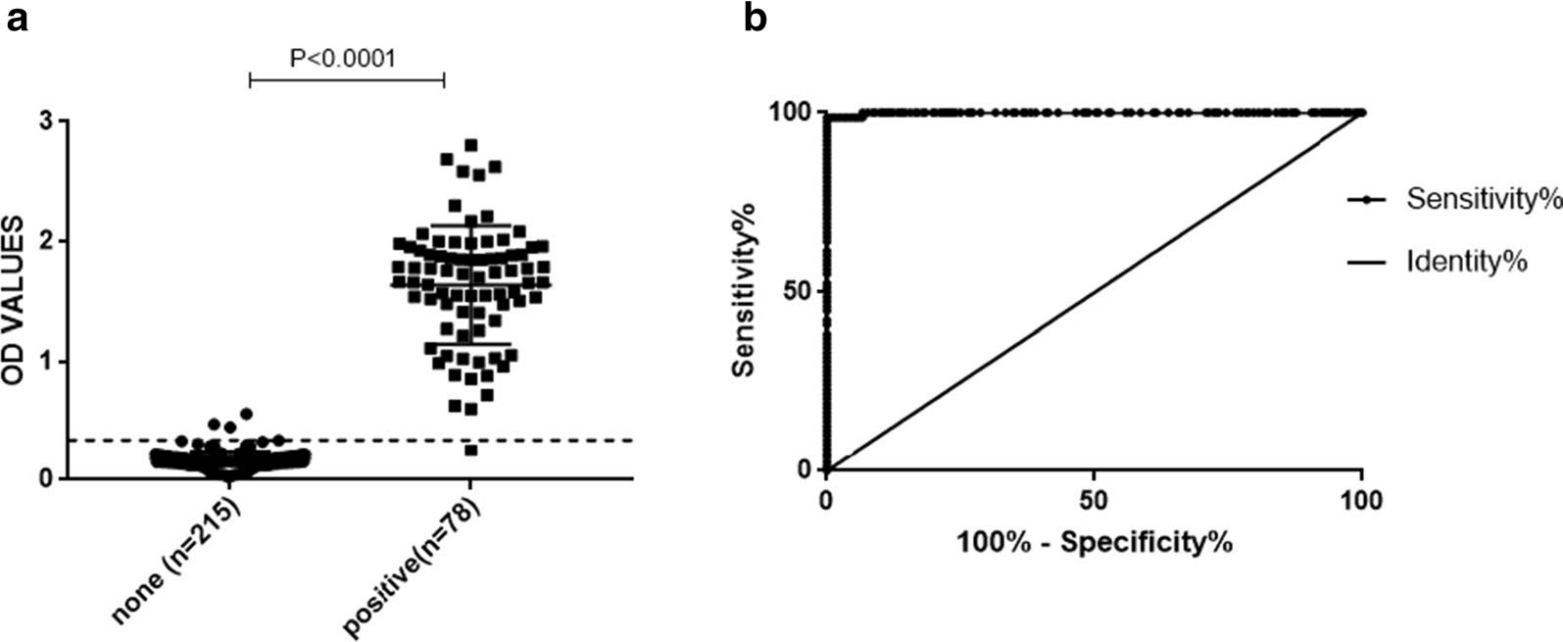
IELISA analysis of serum samples. **a** Dot plot of the m35 iELISA assay. **b** ROC analysis of m35 iELISA assay results. The results of the ELISA, confirming 98.72% sensitivity and 98.14% specificity

**Table 4:**
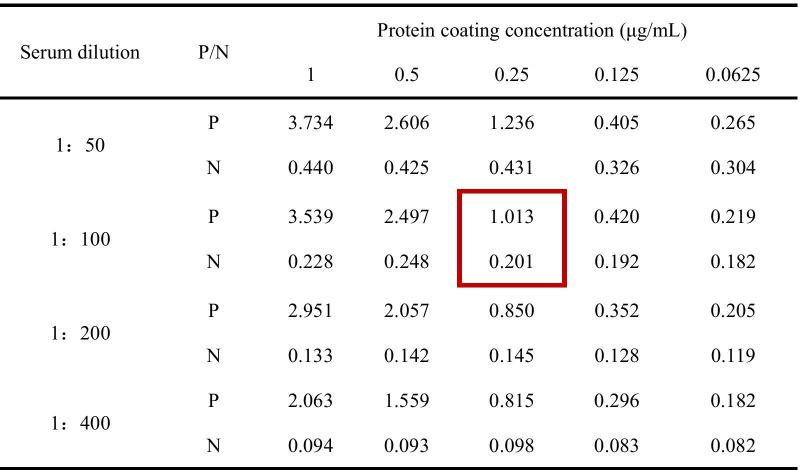
Checkerboard titration results

P: positive serum; N: negative serum. Condition select the maximum P/N value, and the P-value is about 1 from the protein coating concentration and serum dilution such as the red frame chosen

**Table 5:** ASFV negative serum sample test result

Negative serum OD_450/630 nm_							
0.170	0.210	0.170	0.23	0.214	0.177	0.181	0.221
0.165	0.238	0.238	0.222	0.150	0.158	0.340	0.336
0.121	0.220	0.080	0.160	0.218	0.198	0.231	0.248
0.220	0.181	0.181	0.186	0.221	0.236	0.179	0.192
0.213	0.162	0.162	0.217	0.298	0.186	0.236	0.216
0.170	0.140	0.140	0.228	0.231	0.133	0.236	0.179
0.175	0.161	0.161	0.203	0.208	0.166	0.228	0.151

To assess the method's sensitivity, 293 serum samples were tested by indirect ELISA, including positive serum (78 samples) and negative serum (215 samples). The dot plot summarizes the OD values of these samples (Fig. 4). ROC analysis was performed to assess the best sensitivity and specificity. The AUC for this test was 0.9991 (95% confidence interval (CI) 0.9973to 1.001) based on ROC analysis. Also, the diagnostic sensitivity of 98.72% (95% CI, 93.06 to 99.97) and a specificity of 98.14% (95% CI, 95.31 to 99.49) were attained from the optimal cut-off value (0.3380).

It is worth mentioning that under the cut-off value of 0.3380, all ASF carrier pig samples are positive.

### Cross-reaction experiment of ELISA

**Fig. 5:**
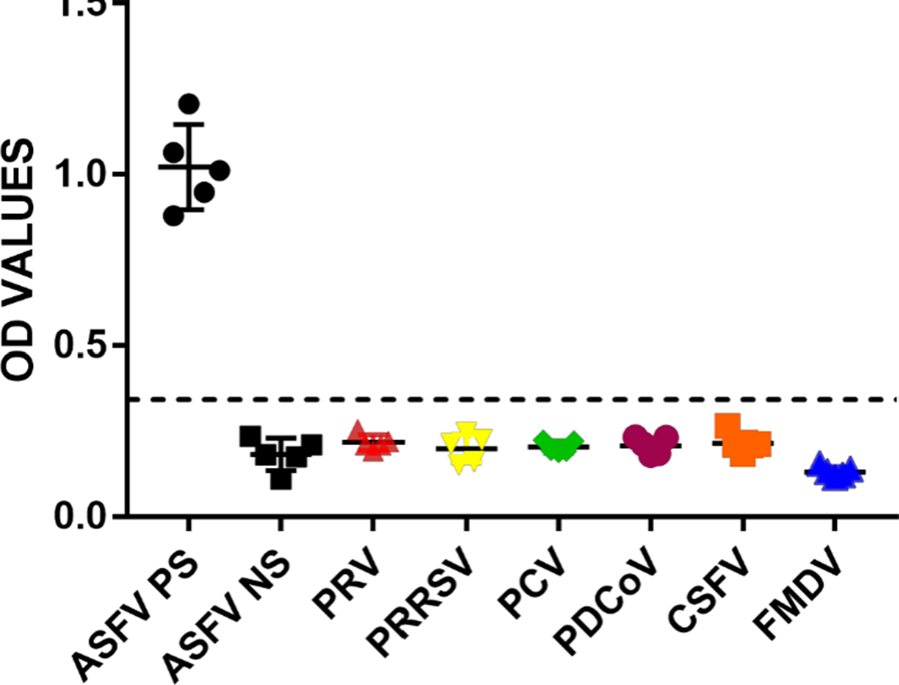
IELISA analysis of other swine disease serum samples. ASFV PS: ASFV positive serum, NS: negative serum, Using PRV, PRRSV, PCV, PDCoV, CSFV, FMDV positive serum test ELISA reaction, OD value is lower than the cut-off value

Evaluating the specificity and sensitivity of ELISA Cross-reaction test was performed on PRV, PRRSV, PCV, PDCoV, CSFV, and FMDV positive sera using established ELISA methods. The results demonstrated that these serum samples were ASFV-seronegative and non-cross-reactive with this m35 indirect ELISA (Fig. 5), indicating that the established ELISA was an effective method for detecting ASFV antibodies.

### B cell epitope identification results

**Fig. 6:**
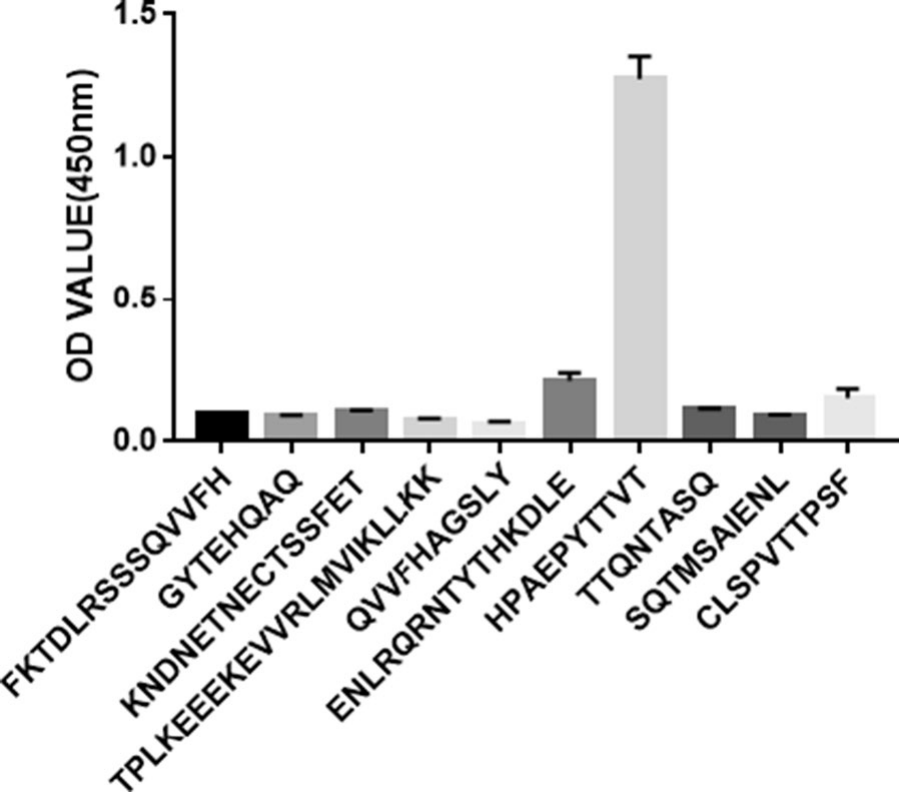
B cell epitope identification results

The kit was used to detect the dominant B cell epitope in the recombinant protein. The results are shown in Fig. 6. The p54 protein sequence 145–151 (HPAEPYTTVT) has a significantly higher reactivity with the African swine fever virus positive serum than other peptides.

## Discussion

The current laboratory diagnostic procedures for ASF include animal vaccination, virus isolation, virus nucleic acid testing, and specific antibody testing. Of these, animal vaccination, virus isolation, and viral nucleic acid detection require professionals who work in laboratories above level 3 [9]. Despite that objective data are obtained, the cumbersome operation limits its wide application, cumbersome operation limits its widespread applications. Although polymerase chain reaction (PCR), Loop-mediated isothermal amplification (LAMP), and recombinase polymerase amplification (RPA) are simple, sensitive, and responsible. Still, it may carry over contaminants in fields and cause false positives [10–14]. Due to its simple procedure and low cost, serological testing is a commonly used diagnostic method for ASFV [15]. It is one of the most important methods for diagnosing and monitoring pigs infected with ASFV [16].

It is not uncommon to use bioinformatics technology to develop vaccines. The tertiary structure of a protein is affected by the secondary structure, and the secondary structure of a protein often affects its potential as an epitope. For example, the α-helix and β-sheet structures of proteins are relatively stable, not suitable for deformation, and it is difficult for chimeric antibodies to become epitopes. However, β-turns and random coils are more likely to protrude on the protein's surface, which is relatively loose and easily twisted., It is easier to become the dominant epitope of B cells. AREGA et al. [17] applied bioinformatics and immunoinformatics methods to computer docking and molecular dynamics simulation of tuberculosis receptors to screen out candidate subunit vaccines that can cause tuberculosis-specific cellular and humoral immune responses. MULPURU et al. [18] identified CTL epitopes of COVID-19 based on immunoinformatics and used sequence conservation studies and molecular dynamics models to identify the epitopes further. SADAT et al. [19] compared and analyzed the protein sequence's structural characteristics and immunogenicity in COVID-19, established a model, and evaluated the MHC-I and MHC-II epitopes with higher antigenicity.

Here we chose P30 and P54 proteins as the target of epitope prediction. Although P30 and P54 have been applied in detecting ASFV antibodies as early as in the last century [20], both P30 and P54 can induce specific immune responses [21]. P30 protein, encoded by CP204L, mainly involves virus internalization and plays an essential role in virus entry into the host cell [22]. Additionally, P30 protein levels are higher in the early stages of viral infection and induce neutralizing antibodies [23]. The P54 protein encoded by the E183L gene is present in the inner envelope of virions and is involved in the virus's adsorption and entry. Therefore, the P30 and P54 proteins are ideal antigens for serological diagnosis and immunological detection [24, 25]. We linked the prediction results to a recombinant protein to test it as a diagnostic antigen. From the two prediction methods in Fig. 2, it can be seen that the recombinant protein has a larger area of antigenicity. The tested recombinant protein had a small molecular weight, was easy to express, and had a lower production cost. After that, we use the checkerboard titration method to determine the best conditions for establishing ELISA and determine the cut-off value by measuring negative serum samples. Then serum samples with a transparent background were used for ROC analysis. The initial results revealed that the antigen had high sensitivity and specificity (0.9 < AUC < 1). After testing, no reaction was noted in the case of other pig diseases. Consequently, it is feasible to use the m35 recombinant protein as a diagnostic antigen to distinguish ASFV infection.

Besides, recent studies have shown that clinically healthy ASFV infection survivors who become carriers can transmit ASFV to pigs on contact [26]. The results of the iELISA method were positive while using survivors' serum samples. Therefore, our method can also play a role in monitoring ASF purification in farms. However, a limitation is that we did not use field serum samples to prove this method's accuracy further. It is worth mentioning that the epitope identified in this study (Fig. 6) P54 protein 145–151 is consistent with the epitope antigen identified in previous reports [27]. However, ELISA identification results by only positive serum and epitope peptides are still a bit weak because some protein sequences in the virus particles cannot be fully exposed due to conformation and other factors, so that the epitope cannot recognize the positive serum. Amino acids 73–81 and 115–123 in the predicted P30 epitope were approximately the same as those in epitopes 61–90 and 116–125, as proven in previous reports. Besides, by comparing the previous report, we can see that in the prediction result of the P30 protein epitope, The prediction results of Scratch and IEDB have covered the epitope reported 116–130 and 146–160. In the P54, Scratch and ABCpred Prediction covers this paper and previously reported advantageous antigenic epitopes. And we can also find the importance of predicting the peptide-SLA-1 binding ability, as NetMHCpan's predictions contain almost all previously declared epitope areas [28, 29].

## Conclusion

In conclusion, we use bioinformatics methods to directly predict and analyze the virus structural protein's amino acid sequence and establish an antibody detection method. Compared with the intact protein’s ELISA method, the gene sequence of the epitope series owns a small molecular weight and is easier to express. Compared with the ELISA method established by traditional epitope proteins, the sequence predicted by bioinformatics saves the time and effort of epitope identification.

## Data Availability

Not applicable.

## Abbreviations

ASF: African swine fever
ASFV: African swine fever virus
CI: Confidence interval
ELISA: Enzyme-linked immunosorbent assay
LB: Luria–Bertani
PVDF: Polyvinylidene fluoride
HRP: Horseradish peroxidase
DAB: Diaminobenzidine
ROC: Receiver-operating characteristic
PRV: Pseudorabies virus
PRRSV: Porcine reproductive and respiratory syndrome virus
PCV: Porcine circovirus
PEDV: Porcine epidemic diarrhea virus
PDCoV: Porcine deltacoronavirus
CSFV: Classical swine fever virus
FMDV: Foot-and-mouth disease virus
PCR: Polymerase chain reaction
LAMP: Loop-mediated isothermal amplification
RPA: Recombinase polymerase amplification
